# Sporadic Burkitt’s lymphoma of the axilla: CT and MR imaging findings

**DOI:** 10.1259/bjrcr.20150481

**Published:** 2016-11-02

**Authors:** Yukihiro Hama

**Affiliations:** Department of Radiology, Tokyo-Edogawa Cancer Center, Edogawa Hospital, Tokyo, Japan

## Abstract

Burkitt's lymphoma (BL) is a high-grade B-cell non-Hodgkin’s lymphoma, which can be divided into endemic, sporadic and human immunodeficiency-associated subtypes. The sporadic BL typically presents as an intra-abdominal tumour but that of cutaneous or subcutaneous origin is very uncommon. We present a case of sporadic BL arising from the axillary soft tissue and discuss the CT and MRI findings.

## Summary

Burkitt’s lymphoma (BL) is a high-grade B-cell non-Hodgkin’s lymphoma (NHL) that often presents with extranodal disease and occurs most often in children and immunocompromised hosts.^1^ The most common site of BL is the abdomen (60–80%), followed by the head and neck region.^1^ BL can be divided into endemic, sporadic (the predominant type found in non-malarial areas) and human immunodeficiency-associated subtypes.^2^ The sporadic type of BL accounts for 1–2% of all adult lymphomas and is seen throughout the world, mainly in children and adolescents, and typically presents as an intra-abdominal tumour.^1,3^ Cutaneous involvement in BL is very uncommon, and only a few cases of imaging findings have been reported in the literature;^4,5^ however, CT and MRI findings of axillary BL have not been documented. BL tends to grow very rapidly, so early diagnosis and treatment is crucial. Here, we present an unusual case of sporadic BL involving the subcutaneous tissue of the axilla and discuss the CT and MRI findings.

## Case report

**Figure 1.**
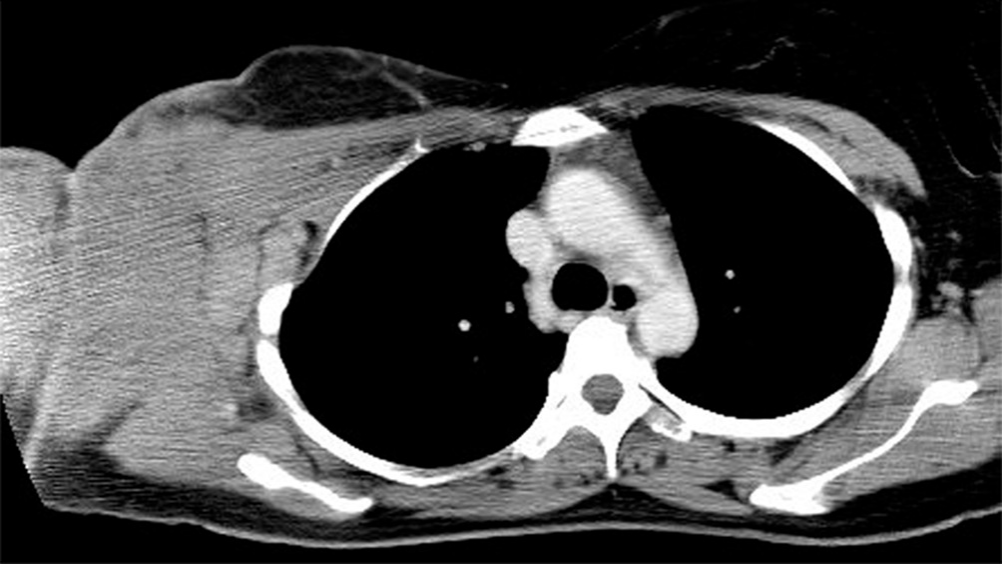
Contrast-enhanced CT scan shows a large, poorly marginated, homogeneous tumour involving the skin and subcutaneous fat. The right axillary lymph nodes are of large size, suggesting lymph node metastases.

**Figure 2.**
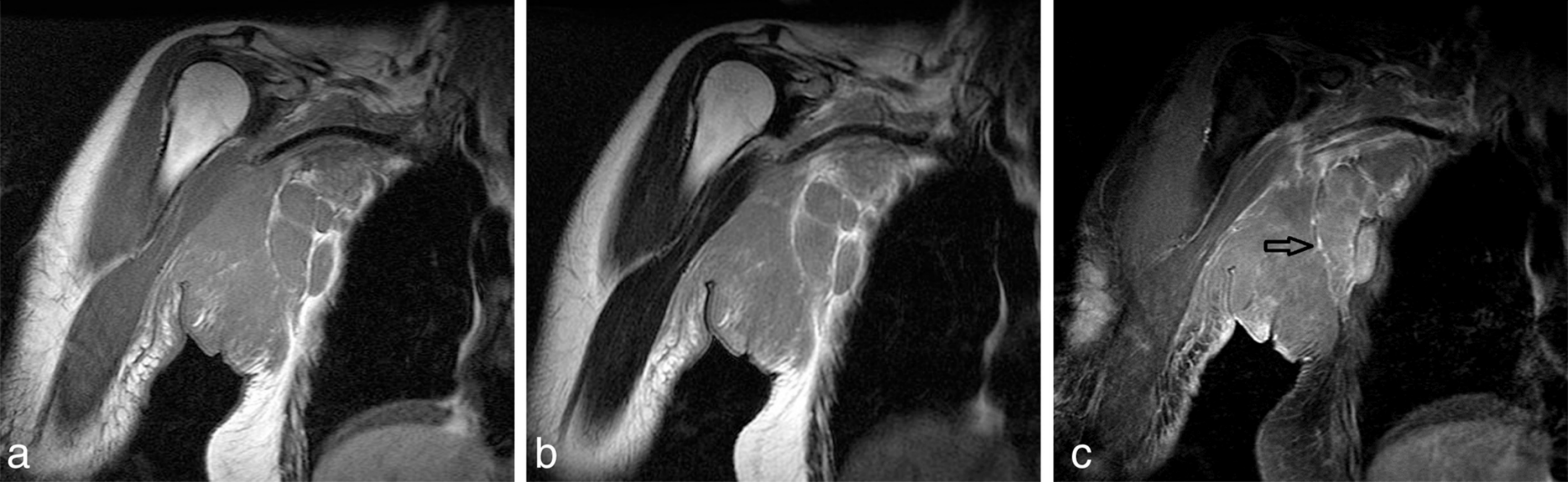
(a) Coronal 2D *T*_1_ weighted SE MRI revealing homogeneous mass of slightly increased signal intensity compared with normal muscle (TR = 659 ms, TE = 20 ms, FA = 90°, SL = 8 mm, matrix = 512 × 512). Coronal 2D *T*_2_ weighted SE MRI showing both the subcutaneous tumour and the axillary lymph nodes had intermediate signal intensity. The lumen of the subclavian artery is intact (TR = 2697 ms, TE = 90 ms, FA = 90°, SL = 8 mm, matrix = 512 × 512). fat-suppressed contrast-enhanced *T*_1_ weighted SE MRI showing diffuse, homogeneous contrast enhancement. Subcutaneous stranding, skin thickening and marginal septal enhancement (arrow) are also present (TR = 659 ms, TE = 20 ms, FA = 90°, SL = 8 mm, matrix = 512 × 512). 2D, two-dimensional; FA, fractional anisotropy; SE, spin echo; SL, slice; TE, echo time; TR, repetition time.

A 33-year-old Asian female patient was referred to our hospital because of rapidly growing subcutaneous nodules in the right axilla. Her medical history was unremarkable, and there was no family history of BL or infection with *Plasmodium falciparum* malaria. The patient initially noticed a small non-pustular nodule in the right axilla. The axillary lymph nodes were not felt at that time. She was initially diagnosed with an atheroma by the referring physician and underwent excision of the skin lesion 4 weeks prior to admission. Pathological examination was not performed. Physical examination revealed a swollen, painful red lump on her right axilla. Complete blood count results were as follows: white blood cells: 6400 μl^−1^; lymphocyte count: 2163 μl^−1^; haemoglobin: 11.3 g dl^−1^; mean corpuscular volume: 88 fl; platelets: 208,000 mm^−3^. Biochemical profile was normal except for elevated levels of serum lactate dehydrogenase 1148 IU l^−1^ (normal: 105–333 IU l^−1^) and C-reactive protein 1.5 mg l^−1^ (normal: < 0.3 mg l^−1^). The patient was seronegative for anti-human immunodeficiency virus antibody and anti-Epstein–Barr virus immunoglobulin M and G. Axial contrast-enhanced CT scan showed a large, poorly marginated homogeneous soft tissue mass in the cutaneous and subcutaneous compartments of the right axilla, and the tumour was slightly enhanced after intravenous administration of contrast medium (Figure 1). MRI of the right axilla was performed to further characterize the lesion using a 1.5 T unit (Signa HDxt 1.5 T, GE Healthcare, Waukesha, WI). Coronal *T*_1_ weighted MRI revealed a homogeneous mass of slightly increased signal intensity compared with normal muscle (Figure 2a). On *T*_2_ weighted MRI, both the subcutaneous tumour and the axillary lymph nodes had intermediate signal intensity (Figure 2b). Fat-suppressed contrast-enhanced *T*_1_ weighted MRI showed homogeneous contrast enhancement (Figure 2c). Skin thickening and marginal septal enhancement were also present. The subclavian vein and artery were not invaded or encased by the tumour. From these findings, the differential diagnoses of this mass were lymphoma, melanoma, breast cancer, fibrosarcoma and malignant peripheral nerve sheet tumour. Excisional biopsy of the axillary mass revealed an enlarged lymph node with “starry sky” appearance owing to abundant macrophages. Immunohistochemical and flow cytometric evaluation showed strong expression of CD20, CD10, CD19, CD22 and surface κ immunoglobulin light chain, but weak or no expression of CD3, Ki-1, bcl-2, cyclin D1 and CD34. Based on these findings, a diagnosis of BL was made. The patient received cyclophosphamide (200 mg m^−2^ day^−1^, days 1–5), doxorubicin (50 mg m^−2^ day^−1^; day 5), vincristine (1.3 mg m^−2^ day^−1^; day 2), dexamethasone (10 mg m^−2 ^ day^−1^ orally; days 1–5, tapered in 1 week), followed by intrathecal chemotherapy with 15 mg of methotrexate and 4 mg of dexamethasone. The patient achieved complete remission and received eight courses of maintenance therapy consisting of cyclophosphamide, doxorubicin, vincristine and dexamethasone. She remains in complete remission at 3 years after the treatment.

## Discussion

In this case, the contrast-enhanced CT scan showed a poorly marginated homogeneous mass in the cutaneous and subcutaneous tissue with axillary lymphadenopathy. These findings were suggestive of a diagnosis of lymphoma or cutaneous infection but were not conclusive. MRI is more sensitive and more specific than CT for the detection and evaluation of soft tissue neoplasms. The tumour had slightly increased signal intensity compared with normal muscle on both *T*_1_ and *T*_2_ weighted MRI, and homogeneous contrast enhancement of the axillary tumour with skin thickening and marginal septal enhancement. These MRI findings are compatible with soft tissue lymphoma.^4,5^ The enhancement pattern seen in this patient may be related to the infiltrative involvement of lymphoma cells and lymphoedema caused by them.^5^ However, considering that lymphoma is rarely infiltrative but normally grows as a well-circumscribed mass, we suspect that the skin thickening and septal enhancement may be more related to lymphoedema as a result of the enlarged lymph nodes, leading to impaired lymphatic drainage. These MRI findings are still inconclusive, yet they may aid in narrowing the differential diagnosis of BL. Since CT and MRI findings of axillary BL have not been reported so far, the current case may help radiologists in diagnosing a patient with soft tissue BL in urgent need of treatment. We consider only after showing real images, one can prove that the imaging findings of soft tissue BL are not different from the probable result.

In summary, the presence of extranodal involvement, particularly in the cutaneous and subcutaneous tissue, together with an aggressive clinical history in a young patient should be suggestive of the possibility of sporadic BL.

## Learning points

1. BL can involve cutaneous/subcutaneous tissues.
2. BL manifestations may show slightly higher signal intensity than muscle on both *T*_1_ and *T*_2_ weighted MRI.

## Consent

Informed consent to publish this case (including images and data) was obtained and is held on record.
